# Connective Adaptive Resistance Exercise (CARE) Machines for Accentuated Eccentric and Eccentric-Only Exercise: Introduction to an Emerging Concept

**DOI:** 10.1007/s40279-023-01842-z

**Published:** 2023-04-25

**Authors:** James L. Nuzzo, Matheus D. Pinto, Kazunori Nosaka

**Affiliations:** Centre for Human Performance, School of Medical and Health Sciences, Edith Cowan University, 270 Joondalup Drive, Joondalup, WA 6027 Australia

## Abstract

Eccentric resistance exercise emphasizes active muscle lengthening against resistance. In the past 15 years, researchers and practitioners have expressed considerable interest in *accentuated* eccentric (i.e., eccentric overload) and eccentric-*only* resistance exercise as strategies for enhancing performance and preventing and rehabilitating injuries. However, delivery of eccentric resistance exercise has been challenging because of equipment limitations. Previously, we briefly introduced the concept of connected adaptive resistance exercise (CARE)—the integration of software and hardware to provide a resistance that adjusts in real time and in response to the individual’s volitional force within and between repetitions. The aim of the current paper is to expand this discussion and explain the potential for CARE technology to improve the delivery of eccentric resistance exercise in various settings. First, we overview existing resistance exercise equipment and highlight its limitations for delivering *eccentric* resistance exercise. Second, we describe CARE and explain how it can accomplish accentuated eccentric and eccentric-only resistance exercise in a new way. We supplement this discussion with preliminary data collected with CARE technology in laboratory and non-laboratory environments. Finally, we discuss the potential for CARE technology to deliver eccentric resistance exercise for various purposes, e.g., research studies, rehabilitation programs, and home-based or telehealth interventions. Overall, CARE technology appears to permit completion of eccentric resistance exercise feasibly in both laboratory and non-laboratory environments and thus has implications for researchers and practitioners in the fields of sports medicine, physiotherapy, exercise physiology, and strength and conditioning. Nevertheless, formal investigations into the impact of CARE technology on participation in eccentric resistance exercise and clinical outcomes are still required.

**Supplementary Information:**

The online version contains supplementary material available at 10.1007/s40279-023-01842-z.

## Key Points

**Table:** 

Delivery of eccentric resistance exercise is often unfeasible because of mechanical and practical limitations of free weights, weight stack machines, and plated-loaded machines
Connected adaptive resistance exercise is the integration of software and hardware to provide a resistance that adjusts in real time and in response to the individual’s volitional force within and between repetitions
Connected adaptive resistance exercise technology can deliver accentuated eccentric and eccentric-only resistance exercise safely and feasibly and thus has the potential to increase participation in, and dose potency of, eccentric resistance exercise in multiple settings and in various populations

*“[O]bviously of some concern is the practicality of [eccentric] exercise. To date, the equipment for the practice of eccentric contractions which the writer has seen has been excessively heavy, cumbersome, and expensive. It is evident that if negative exercise is ever to receive popular acceptance, a great deal of study will have to be given to ways to developing more practical apparatus.”* Dr. Phillip J. Rasch, early resistance exercise researcher (1974) [1].

## Background

Eccentric resistance exercise emphasizes active muscle lengthening against resistance or load. In the past 15 years, researchers and practitioners in sports medicine, physiotherapy, exercise physiology, and strength and conditioning have expressed considerable interest in *accentuated* eccentric (i.e., eccentric overload) and eccentric-*only* resistance exercise and ways to improve the delivery of these modes of exercise [2–20]. Interest in eccentric resistance exercise stems from evidence that (a) humans can generate approximately 40% more force during eccentric than concentric muscle actions (although this difference depends on age, joint action, and movement velocity [21]); (b) cardiovascular stress and perceived effort are lower during eccentric than concentric exercise at equal workloads [22–27]; and (c) weeks of eccentric resistance exercise increase muscle size and strength and improve various clinical outcomes [2, 7, 8, 10, 28–30].

Practitioners often prescribe eccentric resistance exercise. Of strength and conditioning coaches, 67–98% prescribe it, with injury prevention and rehabilitation the most frequently cited reasons (Table 1). However, various factors hinder practitioners from prescribing eccentric resistance exercise more regularly. One factor is equipment. Twenty-three percent of strength and conditioning coaches say *inadequate equipment* is the most significant barrier to implementation of eccentric resistance exercise [31]. Lack of relevant equipment also appears to be an issue for rehabilitation practitioners [9, 14]. Thus, both strength and conditioning coaches and physiotherapists could benefit from new technologies that overcome the limitations of resistance equipment that make eccentric resistance exercise unfeasible (see Sect. 2). In fact, *technology* has been cited by strength and conditioning coaches as the most important future trend in their profession and the most important way in which exercise prescriptions can be improved [32].

**Table 1:** Summary of surveys that have reported practitioner use of eccentric resistance exercise

Reference	Sample	Findings related to use of eccentric resistance exercise
Chesterton and Tears 2021 [93]	17 physiotherapists and S&C practitioners from various countries who coach professional cricketers	For hamstring injury prevention, 100% of practitioners prescribe Nordic hamstring resistance exercise, 100% prescribe other eccentric resistance exercise, 67% prescribe concentric resistance exercise, and 8% prescribe isokinetic resistance exercise
Chesterton et al. 2020 [94]	53 physiotherapists, physicians, and S&C practitioners working with English professional soccer clubs	Of practitioners who implement hamstring injury prevention programs, 95% prescribe Nordic hamstring resistance exercise, 93% prescribe other eccentric resistance exercise, 72% prescribe concentric resistance exercise, and 26% prescribe isokinetic resistance exercise
Chesterton et al. 2022 [95]	24 physiotherapists, physicians, and S&C practitioners working with Major League Soccer clubs	Of practitioners who implement hamstring injury prevention programs, 94% prescribe other eccentric resistance exercise, 89% prescribe Nordic hamstring resistance exercise, 83% prescribe concentric resistance exercise, and 33% prescribe isokinetic resistance exercise
Drury et al. 2021 [31]	64 S&C practitioners from various countries who coach youth athletes	(a) 96% of S&C coaches agree or strongly agree eccentric resistance exercise is important for youth athletes; (b) injury prevention is the most common reason for prescribing eccentric resistance exercise to youth athletes; (c) 100% of S&C coaches believe eccentric resistance exercise is important or highly important for hamstring training in youth athletes; (d) equipment used by S&C coaches for eccentric resistance exercise includes body weight (92%), free weights (73%), flywheel machines (16%), and “machine” (16%); (e) perceived barriers to implementation of eccentric resistance exercise include schedule (33%), equipment (23%), training age (17%) knowledge (9%), safety (8%)
Harden et al. 2020 [96]	100 S&C practitioners who coach national- or international-level athletes	(a) 75% of S&C coaches prescribe accentuated eccentric resistance exercise; (b) > 90% of S&C coaches prescribe eccentric resistance exercise for injury prevention and rehabilitation; (c) equipment used by S&C coaches for eccentric resistance exercise includes free weights (~ 55%), plate-loaded machines (~ 50%), flywheel machines (~ 30%), cable machines (~ 25%), “releasers” (~ 10%), isokinetic dynamometers (~ 10%), eccentric-specific machines (~ 10%), bands (~ 5%), and manual resistance (~ 1%); (d) ~ 63% of S&C coaches prescribe eccentric loads equal to 100–120% of concentric 1RM (e) 57% of S&C coaches who do not prescribe eccentric resistance exercise cite equipment access as the main reason
Loturco et al. 2022 [97]	49 S&C practitioners from Brazil who coach elite soccer players	67% of S&C coaches prescribe eccentric resistance exercise
McCall et al. 2014 [98]	27 sport science staff, 9 physiotherapists, and 8 medical doctors at professional soccer clubs in various countries	(a) 79.5% of premier league clubs prescribe eccentric resistance exercise to prevent non-contact injuries; (b) eccentric resistance exercise cited as the most effective exercise to prevent non-contact injuries
McCall et al. 2020 [99]	21 practitioners from various countries who coach elite soccer players	(a) 100% of practitioners agree eccentric resistance exercise is effective at preventing muscle injuries; (b) eccentric resistance exercise is cited as the second most effective training method for preventing muscle injures
McNeill et al. 2020 [13]	98 S&C practitioners from various countries who coach athletes from various sports	(a) 98% of S&C coaches prescribe eccentric resistance exercise; (b) academic journals are the most popular source for information about eccentric resistance exercise among S&C coaches (22%); (c) injury prevention and rehabilitation is the most common reason for prescribing eccentric resistance exercise among S&C coaches (32%); (d) muscle strength (34.9%), hypertrophy (19.2%), power (17.7%), speed (14.1%), flexibility (10.1%), and other (4%) are the specific outcomes targeted by S&C coaches with eccentric resistance exercise; (e) 75% of S&C coaches do not use eccentric strength tests to assess physical performance; (f) the most highly ranked eccentric exercises were bench press (64% of S&C coaches), rear foot elevated lunge (13%), Nordics (11%), and pull ups (9%); (g); equipment used by S&C coaches for eccentric resistance exercise includes free weights (48%), body weight (19%), cable/pulley machines (10%), plate-loaded machines (9%), flywheel machines (7%), and isokinetic dynamometers (2%)
Pote et al. 2016 [100]	24 S&C practitioners from South Africa who coach high school or university cricketers	7% of S&C coaches prescribe eccentric resistance exercise for injury prevention
Weldon et al. 2021 [101]	52 S&C practitioners from various countries who coach professional soccer players	98% of S&C coaches prescribe eccentric resistance exercise
Weldon et al. 2021 [102]	36 S&C practitioners from various countries who coach professional cricketers	91% of S&C coaches prescribe eccentric resistance exercise
Weldon et al. 2022 [32]	55 S&C practitioners from various countries who coach athletes from various sports	(a) 92% of S&C coaches prescribe eccentric resistance exercise; (b) 40% of S&C coaches cite technology, and 8% cite equipment/space, as the way they would modify their practices if they had more time and resources
Zabaloy et al. 2022 [103]	35 S&C practitioners from Argentina who coach elite rugby players	83% of S&C coaches prescribe eccentric resistance exercise

*1RM* one-repetition maximum, *S&C* strength and conditioning

Therefore, the purpose of the current paper is to introduce a new resistance exercise concept and technology called connected adaptive resistance exercise (CARE). First, we overview existing resistance exercise equipment and highlight current limitations in delivering eccentric resistance exercise with such equipment. Second, we define CARE and explain how it is a novel and feasible approach to accentuated eccentric and eccentric-only resistance exercise. We supplement this discussion with preliminary data collected on a CARE machine, expanding on our previous letter [33]. Finally, we discuss the potential for CARE technology to deliver eccentric resistance exercise in various settings and for various populations.

## Equipment: Past and Present

Over the past 160 years, various equipment has been invented to transfer external resistances onto skeletal muscles for resistance exercise [34, 35]. Each equipment has advantages and disadvantages [19, 34, 36–38]. Some disadvantages might be mechanical, such as the way the resistance is transferred to the body. Other disadvantages might be practical, such as machine size, weight, and cost. Below, we focus on mechanical aspects, and we refer readers elsewhere for further discussions on practical considerations of various resistance exercise equipment [19, 34–38].

### Free Weights and Plate-Loaded Machines

Free weights and plate-loaded machines are two of the most frequently prescribed types of equipment for eccentric resistance exercise (Table 1). Because this equipment is gravity dependent and the external load remains constant throughout exercise, it has limitations. First, the constant external loads of free weights and plate-loaded machines are often^1^ lifted without mechanical arrangements that vary their external moments to accommodate for differences in human force-generating capacity throughout movement [39]. Second, constant loads do not accommodate for differences in force-generating capacity between the eccentric and concentric phases of an exercise [40–43]. This hinders both assessments of maximal eccentric strength and prescriptions of eccentric resistance exercise. To deliver an accentuated load in the eccentric phase, practitioners sometimes use “releasers” to dispose of a proportion of the load from a barbell or machine after the eccentric phase has been completed to allow the individual to perform the concentric phase with less load [20]. However, releasers can be difficult to use beyond the first repetition. Moreover, to deliver eccentric-*only* exercise, a manual technique called “negatives” is sometimes used. With negatives, the individual executes the eccentric or negative phase, but “spotters” lift the load in the concentric phase [44]. Negatives are inconvenient because they require spotters who are strong enough to lift the load. Third, with free weights and plate-loaded machines, the load cannot be easily adjusted by the individual once exercise has commenced. Consequently, exercise stops when muscle force is less than what is required to lift the load through the “sticking point” of the concentric phase. This repetition failure, however, is premature in two ways: concentric muscle actions can continue with *lighter* loads (“drop setting”) and eccentric-only actions (negatives) can continue with the same load, if the individual does not need to lift the load in the concentric phase to the start position for the next eccentric muscle action. Drop setting is somewhat inconvenient with such equipment because weight plates need to be removed throughout exercise. Thus, to overcome the issue of concentric phase failure and the inability to automatically reduce load, “forced repetitions,” where a spotter helps the individual perform concentric muscle actions, are sometimes practiced [44].

### Weight Stack Machines

Weight stack machines with cams advanced free weights by providing resistive torques that *vary* through the motion and attempt to match muscle force-generating capacity at different muscle lengths [34, 38, 45, 46]. However, similar to free weights, most cam-based weight stack machines do not provide an eccentric overload. Shapes of cams also are not personalized for individual variations in human “strength curves” [39], and their resistive torque profiles sometimes do not match human strength curves [47–50]. In many cases, these machines are also limited by offering only one exercise per equipment unit.

### Elastic Bands

Elastic bands provide a variable resistance based on the band’s elastic properties [38]. Less than 5% of strength and conditioning coaches prescribe elastic bands for eccentric resistance exercise (Table 1). Elastic bands do not accommodate for differences in eccentric and concentric muscle strength [40–43]. Moreover, their resistive torque profiles are mostly linear: they induce larger resistances at the start of the eccentric phase, when the band is most stretched, and lower resistances at the end of the eccentric phase, when the band is more slack [38, 51]. This pattern of resistive torque does not match many human strength curves [38, 51].

### Body Weight

Body weight is a gravity-dependent form of resistance that is sometimes prescribed for eccentric resistance exercise (Table 1). One issue with body weight resistance is that body weight is not adjustable, though modifications in body posture can alter resistive torques. Nevertheless, body weight resistance will often be either too low or too high for eccentric muscle actions, depending on the exercise and the individual’s strength-to-body mass ratio.

### Isokinetic Machines

With the aforementioned equipment, individuals often displace loads at non-constant velocities. However, this means volitional force throughout movement is not maximal because force is velocity dependent. Isokinetic technology, which was patented in the 1960s and later introduced in academic journals in the same decade [35, 52, 53], was developed to overcome such phenomena by moving limbs at (mostly) constant velocities, allowing for maximal forces to be generated throughout the eccentric and concentric phases. Nevertheless, isokinetic machines have not been widely adopted for eccentric resistance exercise among strength practitioners (Table 1). In addition to practical issues of size and cost, isokinetic machines control only velocity and not load. Thus, to experience eccentric overload, the individual must give maximal or near-maximal effort; otherwise, their limb will be moved passively. Another limitation of isokinetic machines is that they typically only permit single-joint exercises and movement within one degree of freedom. Advantages of these machines are that they record torque in real time, provide visual feedback, save exercise data, are safe for use in rehabilitation programs [28, 30], and are effective at increasing muscle size and strength [2].

### Flywheels

In 2017, Tinwala et al. [19] highlighted the advantages and disadvantages of machines designed specifically for *eccentric* resistance exercise. Most machines exhibited limitations in terms of size, cost, weight, non-adjustable eccentric loads, number of exercises possible, and numbers of degrees of freedom of movement. The authors noted, however, a greater relative number of advantages versus disadvantages for flywheel machines [19]. Flywheels are gravity-independent. They absorb energy created by the individual during the concentric phase and then dissipate this energy (resistance) during the eccentric phase. Between 7 and 30% of strength and conditioning coaches use flywheel machines for eccentric resistance exercise (Table 1). A large body of literature has examined the effects of flywheel training on muscle size and strength [54–56]. A limitation of flywheel machines is that loading during the eccentric phase depends on concentric phase performance [18]. Consequently, accentuated eccentric loading might not occur, or it might occur only through a limited portion of the eccentric phase [18]. Additionally, because eccentric loading with a flywheel machine is caused by the individual’s volitional effort during the concentric phase, researchers and practitioners are unable to control the load in the eccentric phase.

The inability of practitioners to control the load during flywheel exercise has led to the suggestion of using concentric phase velocity as a means to prescribe inertial loads owing to the relationship between the two variables [57]. However, Martín-Rivera et al. [58] have pointed out that flywheels often use rotatory encoders to measure angular velocity at the axis of rotation rather than at the level of the individual. These rotatory encoders are not ideal for differentiating between the concentric and eccentric phases because the direction of the wheel changes only after a repetition is completed rather than between the concentric and eccentric phases. Consequently, *linear* encoders that measure linear velocity in the concentric phase appear to provide a more valid and reliable method of quantifying load [58]. A final limitation of flywheel machines is that they usually do not permit eccentric-*only* exercise [19], which stems from the fact that energy must be placed into the system via a concentric muscle action for the flywheel to provide eccentric loading.

## CARE as an Emerging Concept

### CARE Overview

CARE is the integration of software and hardware to provide a resistance or load that adjusts in real time and in response to the volitional force generated by the individual (within and between repetitions). Consequently, CARE technology, sometimes called “digital weights” or “intelligent load,” can deliver greater resistances during the eccentric than concentric phase of an exercise and it can alter the resistance at different muscle lengths. CARE machines advance isokinetic machines because CARE machine algorithms have the potential to control both load and velocity, and CARE machines can respond to kinetic and kinematic inputs from the individual in real time (e.g., force, displacement, velocity). Depending on the CARE machine, the above can be accomplished for various exercises.

Multiple CARE machines exist. Basic characteristics of these machines are summarized in Table 2. Each machine has unique advantages and disadvantages. Some machines permit only one or two exercises, whereas others permit over a hundred exercises. Some machines are relatively small and designed for home use, whereas others are bulkier and more appropriate for fitness centers or research laboratories.

**Table 2:** List of CARE machines^a^

Company	Country	Machine name	Size (cm)			Weight (kg)	Total maximum load (kg)	Machine cost^b^ (US$)	Published data
			H	W	L				
ARX^c^	USA	Alpha	168	81	239	297	Not listed	$43,000	[104]
		Omni	228	106	325	309	Not listed	$46,000	
Gymgest^d^	China	Power Station	20	115	52	36	120	Not listed	None found
Kineo^e^	Italy	Leg Press	150	75	320	500	440	Not listed	None found
		Multistation	185	90	305	342	440		
		Pulley & Squat	185	90	185	274	440		
		Leg Pro	185	90	260	365	110		
Symotech^f^	Spain	Dynasystem	Not listed			Not listed	Not listed	Not listed	[105–108]
Tonal^g^	USA	Tonal	129	55	13	68	91	~ $2900	None found
Vitruvian^h^	Australia	Trainer ^+^	12	117	52	38	200	~ $2900	[33, 59, 60], Figs. 1 and 2

*CARE* connected adaptive resistance exercise, *H* height, *L* length, *W* width

^a^This table may not include all CARE machines currently available

^b^Represents the cost of the machine hardware, which may or may not include costs of other features

^c^https://www.arxfit.com

^d^http://en.gymgest.vip

^e^https://www.kineosystem.com

^f^https://www.ineditacomunicacion.es/dynasystem/english.html

^g^https://www.tonal.com

^h^https://vitruvianform.com

Here, our purpose is to illustrate the *potential* of CARE technology to deliver accentuated eccentric and eccentric-only resistance exercise rather than to discuss the particular advantages and disadvantages of each machine. To illustrate the potential of CARE technology for delivering eccentric resistance exercise, we describe one CARE machine (Trainer^+^, Vitruvian, West Perth, Australia) We describe this machine because it is the one we are most familiar with [33, 59, 60] and because it exhibits most features of eccentric exercise technologies recommended by Tinwala and colleagues [19]. For example, the machine has distinct modes for accentuated eccentric and eccentric-*only* exercise, permits multiple degrees of freedom, has an easy-to-use interface via its mobile phone application, and provides real-time performance feedback and options for downloading and sharing results. More specifically, this CARE machine consists of motorized winches that apply forces to two independent ropes that exit the top of the machine’s casing, which the individual stands on for most exercises (e.g., biceps curl, overhead press, squat, deadlift). The winches are controlled by an application on the individual’s smartphone and by software running in the machine. Using a handle or bar attached to the ropes, the individual exerts force against the ropes as the winches retract them. Once exercise has commenced, the machine’s algorithm adjusts loads between 0 and 100 kg per rope and in real time at 50 Hz. The magnitude of the adjustment depends on the individual’s movement velocity, force-generating capacity, and the exercise mode and initial load selected. More specifically, the machine’s algorithm adjusts the resistance based on whether the participant’s movement velocity is above or below certain velocity thresholds, and this is another difference between CARE machines and traditional isokinetic machines, which move the individual’s limb at a constant velocity irrespective of the individual’s force output. With the CARE machine’s default exercise mode, slow movement velocities below the threshold in the concentric phase suggest that the individual is struggling to overcome the target resistance (set before the exercise set begins). When movement velocity is below the threshold, the machine’s algorithm reduces the resistance in real time, permitting the movement velocity to increase and allowing the individual to complete the concentric phase. If the individual’s movement velocity in the concentric phase is above the velocity band, this suggests that the resistance is too light, and the algorithm increases the resistance in real time to make the concentric phase more difficult. In the eccentric phase, slow movement velocities below the threshold cause the algorithm to *increase* the resistance, whereas fast movement velocities cause the algorithm to *decrease* the resistance. Eccentric muscle strength is greater than concentric muscle strength [21]; therefore, the machine delivers an eccentric overload if the individual is giving an effort commensurate with their actual physiological capacity. In the machine’s eccentric-only mode, the algorithm reduces the resistance to zero or near zero in the concentric phase, and it adds resistance at the start of the eccentric phase. The machine also includes a constant resistance mode, where the algorithm does not adjust the resistance throughout the movement or between the eccentric and concentric phases. This mode intends to mimic a free weight load with the exception the mode does not account for inertia.

### CARE in the Laboratory

**Fig. 1:**
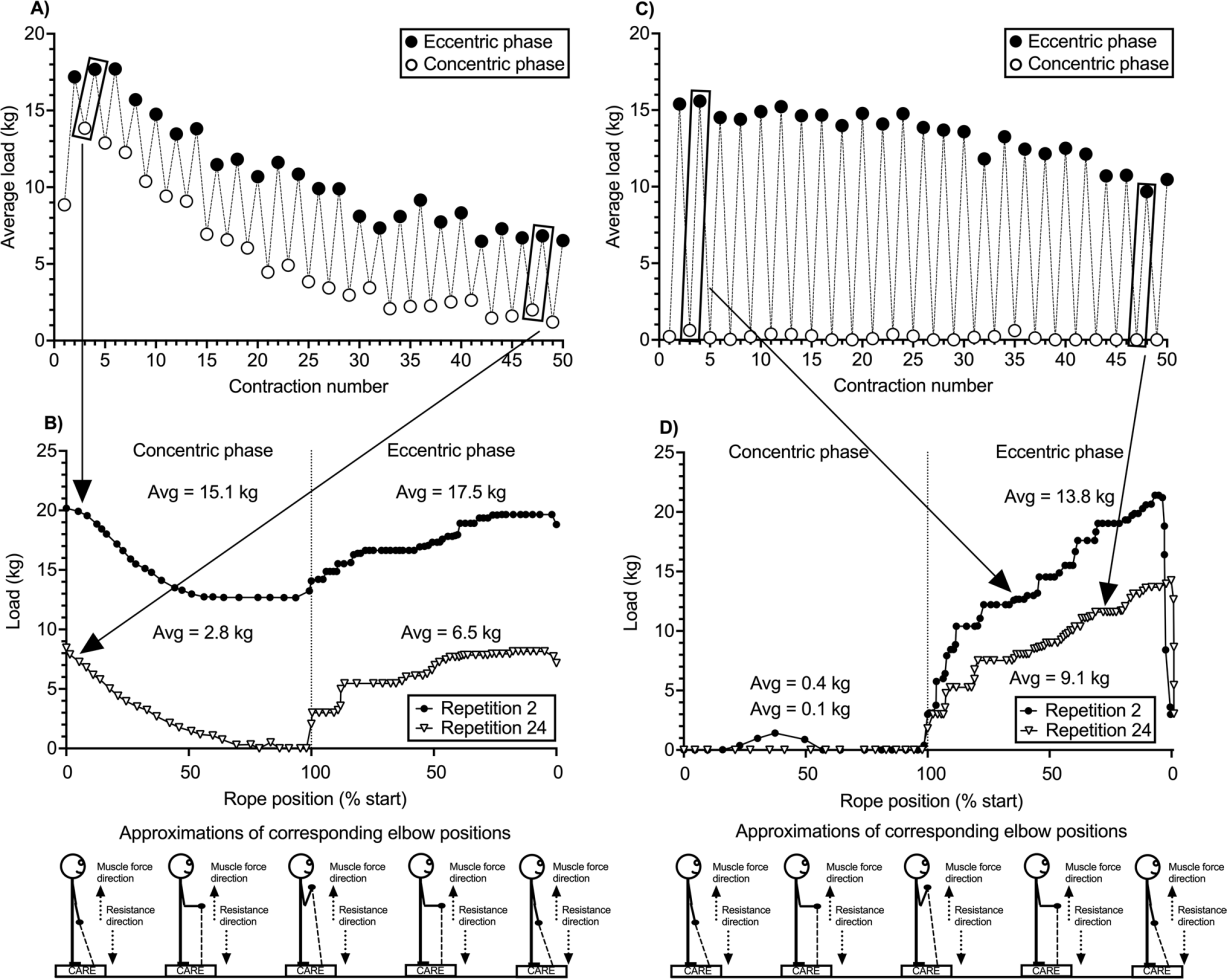
**A** Average (Avg) loads during one set of 25 consecutive maximal effort eccentric-concentric repetitions (50 muscle actions) of the standing unilateral biceps curl performed on a connected adaptive resistance exercise (CARE) machine (Trainer^+^; Vitruvian, West Perth, WA, Australia). The higher Avg loads in the eccentric than the concentric phase reflect the eccentric overload. The reduced Avg loads in the eccentric and concentric phases across the set reflect the automatic drop set feature of the CARE machine in which resistances are reduced as the individual fatigues and loses strength. From repetitions 2 to 24, the individual’s muscle strength reduced 61.4% and 85.5% in the eccentric and concentric phases, respectively. Video 1 of the ESM contains the exercise set from which these data were collected. The csv file from the set, which contains data on load, velocity, and rope position, was exported from the user’s phone, downloaded onto a computer, and imported into Spike2 software (Cambridge Electronics Design, Cambridge, UK) for analysis. The eccentric and concentric phase Avg loads were measured over the middle 50% of the biceps curl movement based on rope position. The middle 50% of the movement range was selected for analysis because it includes the joint angles where elbow flexor torque production is greatest and where the concentric phase sticking point occurs. The low load exhibited in the first concentric muscle action occurred because the algorithm of the CARE machine requires movement before placing load onto the individual. **B** Raw data from repetitions 2 and 24 of the set of 25 eccentric-concentric repetitions depicted in **A**. At the start of the concentric phase, the load was quickly reduced from the preceding eccentric muscle action to match the individual’s reduced strength capacity during the concentric and eccentric phases. Once the machine detected the individual had reached the end of the concentric phase, it increased the load for the eccentric phase. The Avg loads in the eccentric phase were greater than in the concentric phase for repetitions 2 and 24. Substantial reductions in the individual’s muscle strength from repetitions 2 to 24 are obvious based on the downward shift of the load-position curves and can be seen in both the concentric and eccentric phases. Of note, Avg loads displayed for these two repetitions are lower than displayed in panel A because Avg loads from the raw traces were computed from all data points in the respective phases, whereas Avg loads in panel A were computed from the middle 50% of the movement range. These two analysis methods yielded different numbers but similar overall findings. **C** Avg loads during one set of 25 consecutive maximal effort eccentric-*only* repetitions of the standing unliteral biceps curl performed on the CARE machine. The low Avg loads for the concentric phase reflect the machine’s ability to reduce loads in the concentric phase to near zero when using the machine’s eccentric-only mode. Reduced loads in the eccentric phase across the set reflect the automatic drop set feature of the machine. From repetitions 2 to 24, the individual’s muscle strength reduced 37.9% in the eccentric phase. Video 2 of the ESM contains the exercise set from which these data were collected. **D** Raw data from repetitions 2 and 24 of the set of 25 eccentric-*only* repetitions of the standing unliteral biceps curl displayed in panel C. Minimal to no load was placed on the individual during the concentric phase. Once the machine detected the individual had reached the end of the concentric phase, it increased the load for the eccentric phase. The reduction in the individual’s muscle strength from repetitions 2 to 24 is evident from the downward shift in the load-position curve. Of note, Avg loads displayed for these two repetitions are lower than displayed in **C** because Avg loads from the raw traces were computed from all data points in the respective phases, whereas Avg loads in **C** were computed from the middle 50% of the movement range. These two analysis methods yielded different numbers but similar overall findings

Video 1 in the Electronic Supplementary Material (ESM) shows an individual performing 1 set of 25 maximal effort accentuated eccentric (i.e., eccentric-concentric) repetitions of the biceps curl on the CARE machine *in a laboratory environment*. Video 2 of the ESM shows 1 set of 25 maximal effort eccentric-*only* repetitions. Figure 1A,C display average loads during the concentric and eccentric phases for each repetition for the accentuated eccentric and eccentric-only exercise, respectively. Figure 1B,D display load-position traces from repetitions 2 and 24 for the accentuated eccentric and eccentric-only exercise, respectively. Prior to exercise, a load known to be approximately equal to the individual’s eccentric maximum was selected. This resistance reflected the highest load the individual would *ever* experience, not a constant load the subject *always* experienced. The machine adjusted the resistance in real time according to the individual’s movement velocity and force-generating capacity. It delivered higher resistances during the eccentric than concentric phase and decreased the resistance as the individual fatigued. This drop setting feature, first described elsewhere [33], enabled the individual to perform the greatest possible number of repetitions on the machine without need to disengage from it, and this occurred for both the accentuated eccentric and eccentric-only exercise. Two recent experiments [59, 60] have confirmed the automated eccentric overload and drop set features of the machine and also validated the machine’s load in different ways: (a) strong correlations (*r* ≥ 0.94) were found between maximal concentric phase strength on the machine and a one repetition maximum test with a dumbbell; (b) agonist muscle activity during maximal effort repetitions on the machine was equal to or greater than during a one repetition maximum test with a dumbbell; and (c) during fatiguing exercise on the machine, heightened perceptions of fatigue occurred in concert with strength loss.

Data in Fig. 1 illustrate how CARE technology can be used to deliver accentuated eccentric and eccentric-only resistance exercise under supervised conditions. For researchers, CARE technology might resolve several issues. First, in the absence of isokinetic dynamometry, a CARE machine would permit direct measurement of eccentric strength rather than estimation of the eccentric one-repetition maximum and training loads from the *concentric* one-repetition maximum [21]. Second, a CARE machine like the one examined herein would permit examination of neuromuscular adaptations to eccentric resistance exercise interventions comprised of numerous multi- and single-joint exercises. Third, during such research, CARE technology would give the researcher more control over eccentric loading than with machines like flywheels. Fourth, because of the “connected” nature of CARE machines, they could resolve issues of remote data collection in telehealth and sports science research [61] (see also Sect. 3.3) and perhaps allow for a larger scale data collection owing to the automated collection of kinetic and kinematic information from all exercise repetitions.

### CARE in Sport, Fitness, and Rehabilitations Centers

CARE technology offers practitioners a new method for delivering eccentric resistance exercise. Machine algorithms adjust resistances to accommodate changes in an individual’s force-generating capacity within and between repetitions. This advances contemporary resistance exercise equipment described in Sect. 2. With CARE machines, practitioners can deliver an eccentric overload without spotters or releasers. Furthermore, the magnitude of an eccentric overload can be adjusted and delivered for a variety of exercises on some CARE machines. This helps overcome issues of relying on body weight resistance for exercises like the Nordic hamstring exercise. Additionally, if CARE technology makes eccentric resistance exercise more feasible, this might translate to increased effectiveness via more frequent participation in, and more potent doses of, eccentric resistance exercise. The “connected” nature of such technology can allow for data to be stored and then shared among medical staff within sports clubs. The CARE machine examined herein is also relatively small, has wheels to facilitate transport, and allows for numerous exercises. Such equipment might be of value to practitioners who work in small gyms or office spaces, though requirements of electrical power and smartphone access are potential disadvantages.

CARE technology also has the potential to improve aspects of resistance exercise for recreational lifters in fitness centers. For example, negatives and forced repetitions are two resistance exercise methods commonly used by bodybuilders [44]. Both methods require the presence of at least one spotter. With the CARE machine shown herein, negatives (i.e., eccentric-only resistance exercise, Video 2 of the ESM) and forced repetitions (i.e., automatic drop setting, Videos 1 and 2 of the ESM) can be completed without a spotter. Moreover, CARE technology makes drop sets more convenient because the “dropping” occurs automatically as the individual fatigues, and the individual does not need to disengage from the machine to remove bar collars and weight plates. Drop sets are becoming increasingly recognized as an efficient form of resistance exercise because they permit a large volume of resistance exercise to be completed in a short time [62–65]. *Eccentric-only drop sets* could be a new resistance exercise method because of CARE technology.

### CARE for Home Exercise and Telehealth

**Fig. 2:**
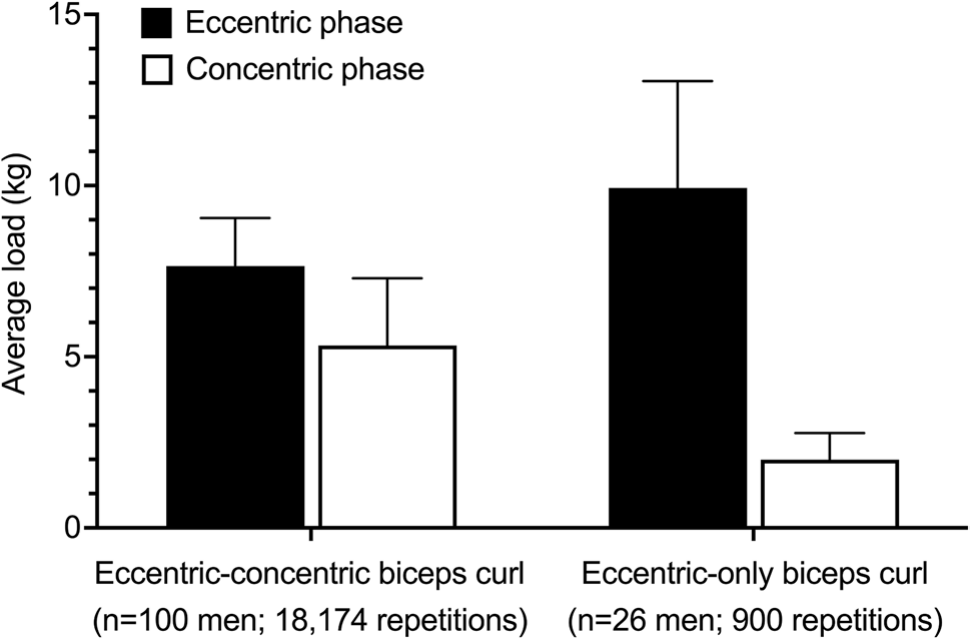
Eccentric and concentric average (± standard deviation) loads from a random sample of 100 men who used a connected adaptive resistance exercise (CARE) machine to perform the biceps curl exercise in non-laboratory environments (Trainer^+^; Vitruvian, West Perth, WA, Australia). Data on the *left* are average loads across 18,174 repetitions performed in 2124 exercise sets (average: 8.6 repetitions/set). These data illustrate that the CARE machine delivered eccentric overload during eccentric-concentric repetitions (eccentric:concentric strength ratio = 1.42). Data on the *right* are average eccentric and concentric loads from 26 of the 100 men who performed a total of 900 eccentric-only repetitions in 135 sets (average: 6.7 repetitions/set). These data illustrate that the CARE machine delivered high loads in the eccentric phase and minimal loads in the concentric phase during eccentric-only repetitions (eccentric:concentric strength ratio = 4.99). All values displayed reflect loads from one arm (i.e., one rope of the machine), irrespective of whether the exercise was performed unilaterally or bilaterally. The average loads are lower than those in Fig. 1 because the data in Fig. 1 reflect maximal effort exercise by one individual, whereas data in the current figure were averaged across several hundred repetitions, and would have been completed at various intensities and levels of fatigue. These data were acquired as part of an ongoing study that has been approved by a university ethics board (#2021-02417-NUZZO)

Some CARE machines are marketed for home use (e.g., Tonal, Vitruvian Trainer^+^). Thus, CARE machines might enhance the effectiveness of resistance exercise performed at home. In Fig. 2, we present evidence of CARE technology delivering accentuated eccentric and eccentric-only resistance exercise *in non-laboratory environments* without supervision. Thus, CARE technology might increase the effectiveness of home-based resistance exercise compared with other equipment, which does not provide an eccentric overload. For example, in older adults, effects of elastic band and bodyweight resistance exercise on muscle strength are small to modest, and the lack of larger effects has been attributed to low external loads, low perceived efforts, and a lack of supervision with home-based resistance exercise [66, 67]. Approaches to home-based eccentric resistance exercise with CARE machines might include minimal effective dose programs, such as “exercise snacking” and “resistance exercise breaks,” where individuals perform low volumes of resistance exercise multiple times each day at moderate-to-high intensities [62, 68–72]. One recent study found that a 3-s maximal *eccentric* muscle action, performed daily for 4 weeks on an isokinetic machine in a laboratory, caused robust improvements in muscle strength, whereas daily maximal concentric and isometric training had minimal effects [73]. Similar eccentric resistance exercise programs could be attempted at home with CARE machines.

Finally, home-based resistance exercise and ways to improve its delivery and effectiveness warrant consideration from a public health perspective given that (a) approximately 30–70% of individuals prefer exercising at home than in other environments [21]; (b) approximately 80% of individuals do not meet guidelines for muscle-strengthening activities [74, 75]; and (c) calls for greater emphasis on resistance exercise in public health have been made [76, 77]. Home-based exercise for public health has also received increased attention because of the COVID-19 pandemic [78]. Pandemic-related policies increased sedentary time at home, decreased physical activity rates [79, 80], and impacted resistance exercise practices [81, 82]. During “lockdowns,” the proportion of individuals performing resistance exercise at home increased from ~ 18% to ~ 89% [82]. This transition in the training environment corresponded with an increased use of body weight resistance, reduced perceptions of effort, and lower exercise “intensities” for many individuals [81, 82]. Perceived effectiveness and enjoyment of resistance exercise, and the likelihood of continuing resistance exercise, were also lower during this time [82]. The pandemic also highlighted the potential importance of telehealth services and delivery of physical exercise to patients who cannot easily access healthcare facilities [83, 84]. It also highlighted issues with remote data collection in sports science [61]. As discussed elsewhere [78], home-based CARE machines could help address such issues, as they appear to provide a stimulus sufficient for neuromuscular adaptation (Figs. 1 and 2), although longer term training studies are required. The “connected” nature of CARE machines might also improve aspects of telehealth interventions and remote data collection. For example, with some CARE technology, individuals can access exercise tutorials, classes, and programs, and their exercise data can be shared with health professionals who wish to monitor adherence and progression.

## Limitations of CARE Technology

We have explained how CARE technology might make the delivery of accentuated eccentric and eccentric-*only* resistance exercise more feasible for exercise practitioners. Nevertheless, the limitations of CARE technology, and other cautionary notes, need to be mentioned. Most importantly, formal investigations into the reliability, validity, and effectiveness of CARE machines for improving muscle size and strength are limited (Table 2). This is in contrast to other types of resistance exercise equipment whose reliability, validity, and abilities to increase muscle size and/or strength are well established [55, 85–90]. Nevertheless, our purpose was to introduce the *concept* of CARE and present preliminary data showing the *potential* of CARE technology to be used for eccentric resistance exercise in various settings [35]. Similarly, James Perrine, the patent holder for the concept of isokinetics [35], introduced the idea of isokinetics in papers published in *Physical Therapy* in 1967 [52] and the *Journal of Health, Physical Education, Recreation* in 1968 [53], with no experimental evidence available at that time. As shown in Figs. 1 and 2 and elsewhere [33], CARE technology can automate the eccentric overload and drop setting, which are training strategies known to increase muscle size and strength [2, 4, 15, 64, 65, 91, 92]. Importantly, we do not propose that CARE machines necessary replace other types of resistance exercise equipment. All equipment has advantages and disadvantages [19, 36, 38], and matching training loads and volumes in scientific studies to determine which equipment induces the *greatest *gains in muscle size and strength is a challenge owing to the different natures of their resistances. Instead, we suggest that CARE technology can be used in conjunction with, or independent of, other resistance exercise equipment, depending on one’s goals, resources, and exercise preferences. We also state that the ability of a machine to allow for accentuated eccentric and eccentric-*only* loads in multiple settings is a noteworthy advance and appears to meet the needs of many coaches and rehabilitation practitioners (Table 1). Future research can explore which types of resistance exercise equipment appeal most to researchers, coaches, athletes, and general consumers.

## Conclusions

Strength practitioners cite *technology* as the most important future trend in their profession and the most important way in which resistance exercise for athletes can be improved [32]. In the current paper, we introduced the concept of CARE and explained how it can be used to achieve accentuated eccentric and eccentric-only resistance exercise in a new way. CARE machines have the potential to mitigate various biomechanical and practical disadvantages associated with contemporary resistance exercise equipment and allow eccentric resistance exercise to be delivered in a safe, feasible, and potentially effective way. Thus, because of their various potential implications for researchers and practitioners in the areas of sports medicine, physiotherapy, exercise physiology, strength and conditioning, and public health, CARE machines might represent a new era of resistance exercise. They might represent the type of apparatus that Rasch [1] had in mind in 1974 when expounding the need for a more practical apparatus to permit greater “popular acceptance” of eccentric resistance exercise. Nevertheless, formal investigation into the impact of CARE technology on eccentric resistance exercise participation and clinical outcomes is still required.

## Supplementary Information

Below is the link to the electronic supplementary material.

Supplementary file1 (MP4 54899 KB)

Supplementary file2 (MP4 59338 KB)

## Footnotes

1 Plate-loaded machines might also be designed in ways that vary their external moments to match strength curves or to vary the external moments in some other purposeful way.
